# Identification and characterization of a novel CASR mutation causing familial hypocalciuric hypercalcemia

**DOI:** 10.3389/fendo.2024.1291160

**Published:** 2024-02-29

**Authors:** Chien-Ming Lin, Yi-Xuan Ding, Shih-Ming Huang, Ying-Chuan Chen, Hwei-Jen Lee, Chih-Chien Sung, Shih-Hua Lin

**Affiliations:** ^1^ Department of Pediatrics, Tri-Service General Hospital, National Defense Medical Center, Taipei, Taiwan; ^2^ Department of Biochemistry, National Defense Medical Center, Taipei, Taiwan; ^3^ Department of Physiology and Biophysics, National Defense Medical Center, Taipei, Taiwan; ^4^ Division of Nephrology, Department of Medicine, Tri-Service General Hospital, National Defense Medical Center, Taipei, Taiwan

**Keywords:** calcium-sensing receptor, familial hypocalciuric hypercalcemia, calcimimetics, parathyroid hormone, half-maximal effective concentration

## Abstract

**Context:**

Although a monoallelic mutation in the calcium-sensing receptor (*CASR*) gene causes familial hypocalciuric hypercalcemia (FHH), the functional characterization of the identified *CASR* mutation linked to the clinical response to calcimimetics therapy is still limited.

**Objective:**

A 45-year-old male presenting with moderate hypercalcemia, hypocalciuria, and inappropriately high parathyroid hormone (PTH) had a good response to cinacalcet (total serum calcium (Ca^2+^) from 12.5 to 10.1 mg/dl). We identified the genetic mutation and characterized the functional and pathophysiological mechanisms, and then linked the mutation to calcimimetics treatment *in vitro*.

**Design:**

Sanger sequencing of the *CASR*, *GNA11*, and *AP2S1* genes was performed in his family. The simulation model was used to predict the function of the identified mutant. *In vitro* studies, including immunoblotting, immunofluorescence, a cycloheximide chase study, Calbryte™ 520 Ca^2+^ detection, and half-maximal effective concentration (EC_50_), were examined.

**Results:**

This proband was found to carry a *de novo* heterozygous missense *I554N* in the cysteine-rich domain of *CASR*, which was pathogenic based on the different software prediction models and ACGME criteria. The simulation model showed that *CASR I554N* mutation decreased its binding energy with Ca^2+^. Human *CASR I554N* mutation attenuated the stability of CASR protein, reduced the expression of p-ERK 1/2, and blunted the intracellular Ca^2+^ response to gradient extracellular Ca^2+^ (eCa^2+^) concentration. The EC_50_ study also demonstrated the correctable effect of calcimimetics on the function of the *CASR I554N* mutation.

**Conclusion:**

This novel *CASR I554N* mutation causing FHH attenuates CASR stability, its binding affinity with Ca^2+^, and the response to eCa^2+^ corrected by therapeutic calcimimetics.

## Introduction

Familial hypocalciuric hypercalcemia (FHH) is an autosomal dominant disorder characterized by a lifelong elevation of serum calcium (Ca^2+^) level with hypocalciuria and inappropriately normal or high parathyroid hormone (PTH) concentrations (1, 2). Based on the causative genetic mutations, FHH type 1 (FHH1; OMIM #145980) caused by a heterozygous loss-of-function mutation in the Ca^2+^-sensing receptor (*CASR*) gene is the most common and estimated to represent approximately 85-90% of all FHH cases, followed by the *AP2S1* gene (FHH3), at approximately 5-10%, and the *GNA11* gene (FHH2), at less than 5% (3–7). Although most FHH patients are usually asymptomatic or mildly symptomatic, e.g., fatigue, weakness, or thought disturbances (8), the increased risk of chronic kidney disease (CKD), coronary heart disease, pancreatitis, femoral fracture, and chondrocalcinosis with advancing age has been reported (9–11). Additionally, patients with FHH may be misdiagnosed as primary hyperparathyroidism, receiving an unnecessary parathyroidectomy, which typically does not successfully normalize hypercalcemia (12).

The majority of the inactivating mutations in the *CASR* gene are missense and are scattered throughout the protein sequence with some clustering in the first half of the extracellular domain (ECD) (venus flytrap (VFT) domain and closely associated with the Ca^2+^-binding sites) and the latter part of the ECD (cysteine-rich region and parts of the transmembrane-spanning region) (**Supplementary Figure 1**) (13). With respect to treatment, calcimimetics act as positive allosteric modulators of the CASR that increases the receptor’s response to extracellular Ca^2+^ (eCa^2+^) levels (8), thus they have been used to treat symptomatic hypercalcemia in certain cases of FHH (14–16). To date, the use of calcimimetics in FHH is still a matter of debate, and the long-term effects of calcimimetic therapy in FHH are not yet fully understood (8). Considering the different mutant sites of the *CASR* gene for biodiversity, the comprehensive identification and characterization of *de novo* variants in CASR will provide valuable insights into the pathogenesis and elucidate the role of calcimimetics in the treatment of FHH (17).

We have encountered an adult FHH patient with moderate hypercalcemia, hypocalciuria, inappropriately high PTH levels, and progressive deteriorated renal function who had a good response to 25 mg oral cinacalcet daily for 6 months. In this study, our objective was to identify his responsible genetic mutation and assess the functional analysis of the identified mutation in relation to calcimimetic response. The results revealed a *de novo* heterozygous mutation (c.T1661A, *I554N*) in the cysteine-rich domain of the *CASR* gene responsible for his FHH. This missense mutation disrupted the binding of CASR I554N with eCa^2+^ in simulation models, leading to a decrease in the stability of CASR protein, a reduction in the expression of p-ERK 1/2, and a diminished response to eCa^2+^ concentration. The half-maximal effective concentration (EC_50_) study also demonstrated the correctable effect with the use of calcimimetics.

## Materials and methods

### Human subjects

This study followed the tenets of the Declaration of Helsinki and was approved by the Ethics Committee of the Institutional Review Board of the Tri-Service General Hospital (TSGH), National Defense Medical Center (TSGHIRB No.:A202105016). All methods were performed in accordance with approved guidelines. Written informed consent was obtained from the participants after a detailed description of the study.

### Index case

The 45-year-old man was referred because of hypercalcemia of unknown causes for more than 5 years and a progressive decline in the estimated glomerular filtration rate (eGFR). There were no obvious symptoms related to hypercalcemia, but easy fatigue, constipation, and worsening renal function were noticed. Family and personal histories were unremarkable. The most striking laboratory abnormality was hypercalcemia (12.5 mg/dL; range, 8.6-10.2) with hypocalciuria (spot urine Ca^2+^/Cr ratio 0.019 mg/mg, 24-hour urinary Ca^2+^ excretion 32 mg/day) and an inappropriately increased iPTH (65 pg/mL; range, 10.0-69.0) (**Table 1**). A sonography of the parathyroid gland and abdomen were normal. Under the diagnosis of FHH, an allosteric modulator of the CASR, oral cinacalcet (25 mg/day), was given. It reduced his serum Ca^2+^ concentration to 10.1 mg/dL and iPTH level to 25 pg/mL, coupled with an increased urine Ca^2+^ excretion (spot urine Ca^2+^/Cr ratio of 0.06) and improved eGFR after the use of calcimimetic agent 6 months later.

**Table 1:** Blood and urine data before and after cinacalcet treatment.

	Normal ranges	Before	After
**Blood biochemistries**			
BUN (mg/dL)	7-25	16	15
Cre (mg/dL)	0.7-1.2	1.1	0.8
eGFR (mL/min/1.73m^2^)	100-120	76	111
Sodium (mmol/L)	136-145	139	138
Potassium (mmol/L)	3.5-5.1	4.3	4.1
Chloride (mmol/L)	98-107	102	104
Total Ca^2+^ (mg/dL)	8.6-10.2	12.5	10.1
P (mg/dL)	2.7-4.5	2.6	2.8
Mg^2+^ (mg/dL)	1.7-2.55	2.3	2.2
iPTH (pg/mL)	10.0-69.0	65	25
**Urine biochemistries**			
Ca^2+^/Cr (mg/mg)		0.019	0.060
FE_Ca_^2+^ (%)		0.17	0.48

BUN, blood urea nitrogen; Cre, creatinine; Ca^2+^, calcium; P, inorganic phosphorus; Mg^2+^, magnesium; iPTH, intact parathyroid hormone; FE_Ca_^2+^ represents the fractional excretion of Ca^2+^.

### Molecular screening of FHH and confirmation of CASR mutation

Peripheral blood was collected and deoxyribonucleic acid (DNA) extracted following a classic phenol–chloroform protocol (QIAamp Blood Kit; Qiagen, Dusseldorf, Germany). Molecular screening of the entire *CASR*, *GNA11*, and *AP2S1* coding sequences (18 exons–21 amplicons, including exon–intron boundaries) was performed. Furthermore, *CASR* mutation was confirmed by sequencing in both directions on the original amplicon and on a different polymerase chain reaction (PCR) product. Nine primer pairs were used to amplify exons 2–7 (which encode the receptor protein) of the CASR gene, as described previously (18). Forward and reverse primers were modified at their 5′-ends by the addition of a T7 or T3 promoter sequence, respectively, to aid in the subsequent nucleotide sequencing of the PCR product.

### Simulation models of CASR mutation

The resolved structures of the extracellular domain of human CASR (PDB code: 5K5S and 5K5T) were used as a template to build the models of WT-CASR using the Homology Modeling protocol (Biovia Discovery Studio 2019). The simulation includes the loop refinement at a high optimization level. The mutant I554N model was generated using the Built Mutants protocol followed by energy minimization. The geometries of the models were optimized using the algorithm of smart minimization in the CHARMM force field including the generalized born implicit solvent model in the calculation.

### Evaluating the effect of the change in mutation energy caused by mutations on the stability of CASR

AlphaFold is a computational method for predicting protein structures with atomic accuracy, even in cases in which no similar structure is known. Additionally, it can be used to evaluate and compare the similarity of these two predictive models and then utilize the results in additional advanced bioinformatic analyses. The effect of residue substitution on the stability of CASR was determined from the predictive Alphafold model using Discovery studio visualizer version v19.1.0.18287 (BIOVIA, San Diego, CA, USA). As for the calculation of the change in mutation, the energy is normalized to CASR-WT.

### cDNA expression vectors and mutagenesis

The *CASR* variants of interest were introduced into a pCMV6 vector expressing Myc-DDK-tagged human WT CASR cDNA (RC211229, OriGene) through site-directed mutagenesis (QuikChange, Stratagene, La Jolla, CA, USA) and confirmed by DNA sequencing analysis. Briefly, the mutagenesis reaction was carried out to generate mutant pCMV6-CASR **I554N**-Myc-DDK and pCMV6-CASR **R220W**-Myc-DDK constructs using the following primers: p. I554N, For: 5′-GCAGGGACCAGGAAAGGG **AAC** ATTGAGGGGGAGCCCACC-3′ and Rev: 5′-GGTGGGCTCCCCCTCAATGTTCCCTTTCCTGGTCCCTGC-3′; p. R220W, For: 5′- GCTGATGACGACTATGGG **TGG** CCGGGGATTGAGAAATTC-3′ and Rev: 5′- GAATTTCTCAATCCCCGGCCACCCATAGTCGTCATCAGC-3′, the mutated bases are underlined).

### Cell culture, plasmid transfection, and protein stability assay

HEK-293 cells were cultured in Dulbecco’s modified Eagle’s medium supplemented with 10% (v/v) fetal bovine serum, 2 mM L-glutamine, 100 U/ml penicillin, and 0.1 mg/ml streptomycin at 37°C in a humidified 5% CO_2_ incubator. HEK-293 cells (4 × 10^5^ cells per 6-well plate) were transfected with the indicated amount of plasmid DNA using Lipofectamine 3000 reagent (Invitrogen). For each transfection, 5 µg of expression vectors was used, and the total amount of plasmid DNA was adjusted by adding empty vectors. Cells were visualized using a fluorescence microscope (Carl Zeiss, Inc., Oberkochen, Germany) with an epifluorescence filter, and images were captured using Openlab software (Improvision Inc. Lexington, MA, USA).

For protein stability analysis, transfected cells were treated with 50 mg/mL cycloheximide (CHX) for the indicated time and harvested for IB, which was performed using mouse anti-Myc monoclonal antibody (TA150121-1; OriGene). Phospho-ERK1/2 (Thr202 and Tyr204) antibody (#9101; Cell Signaling Technology) was used for IB.

### Fluorescence measurements of iCa2+ in the whole cell population

The iCa^2+^ was measured in CASR-expressing HEK293 cells (approximately 5×10^5^ cells/ml for each experiment). HEK293 cells were loaded with 10 mM Calbryte™ 520 (NC1424566; Fisher Scientific), incubated for 120 min in a 5% CO_2_ incubator at 37°C, as described previously (19). The dye loading solution was removed, and fresh cell culture medium was added to the plate. To study the response of mutant CASR variants to eCa^2+^ stimulation, the eCa^2+^ was increased stepwise by the addition of CaCl_2_ at concentrations between 0 mmol/l and 10.0 mmol/l. The cells were collected at the indicated eCa^2+^ concentrations and washed once with Ca^2+^- and magnesium-free Hanks’ balanced salt solution (HBSS) (Invitrogen) before analysis.

For the *in vitro* rescue study, the calcimimetic NPS R-568 (SI-SML2160; Sigma-Aldrich) was added at a concentration range of 1.0 to 100 μmol/l in the presence of the same CaCl_2_ concentration (0.6 mmol/l) (20). The iCa^2+^ was calculated from the ratio of the fluorescence emission recorded at the two-excitation wavelengths (19). The EC_50_ (i.e., iCa^2+^ required for 50% of the maximal response) for each normalized concentration–response curve was determined.

### Statistical analysis

The results were presented as the mean ± standard deviation (SD) for continuous variables. Comparisons between groups were carried out using the Wilcoxon test or χ2 test as appropriate. The mean EC_50_ was calculated, and statistical analysis performed using the Mann–Whitney U test. All analyses were performed using SPSS 20.0 for Windows (SPSS, Chicago, IL). A *p*-value less than 0.05 was considered statistically significant.

## Results

### Identification of a novel *CASR I554N* mutation

**Figure 1:**
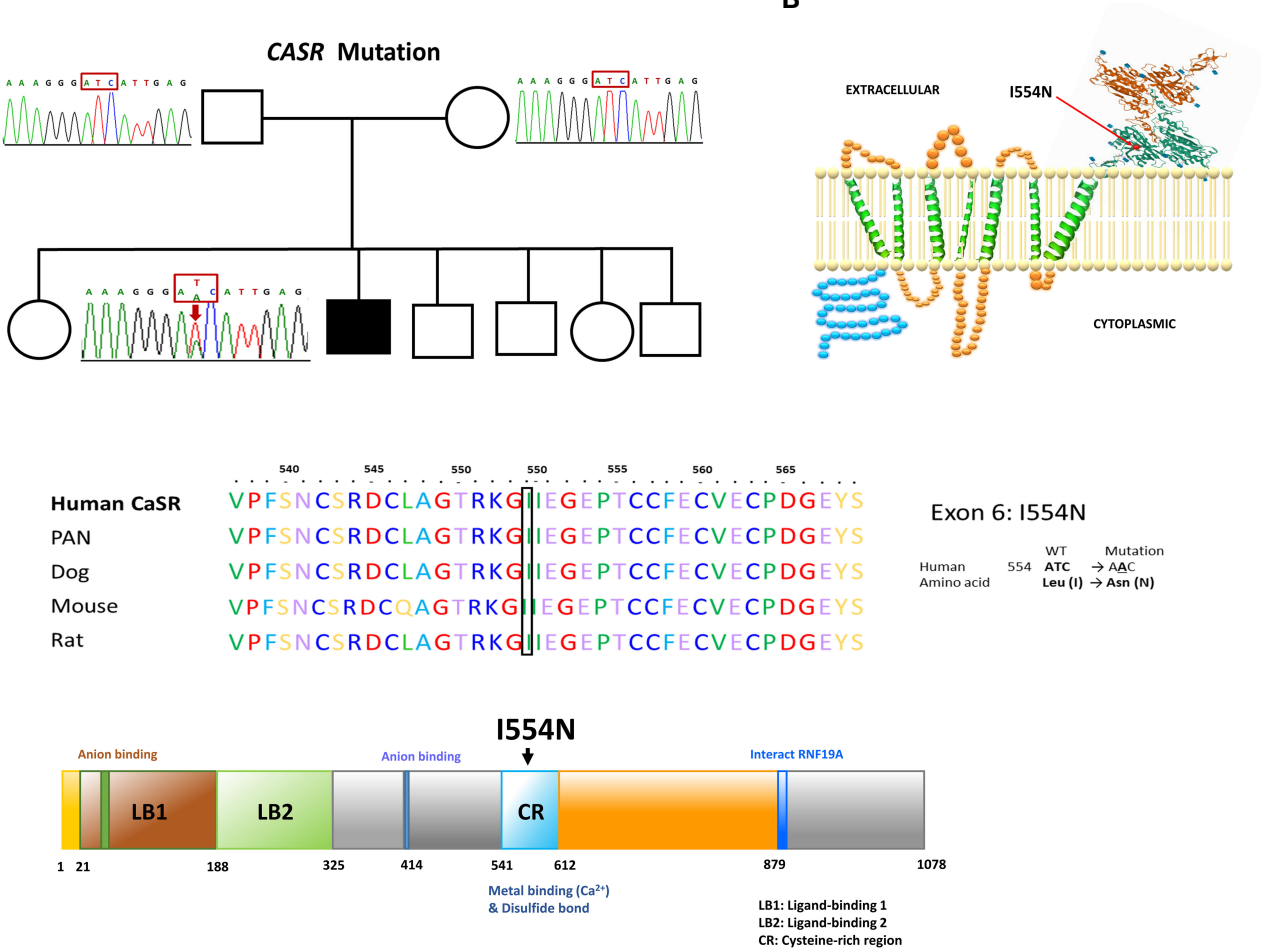
Our FHH1 patient with a *de novo I544N* mutation localized the cysteine-rich region of CASR. **(A)** Pedigree of the family. The squares and circles indicate male and female, respectively. The black shading indicates the affected FHH1 patient. Sanger sequencing identified the heterozygous *de novo* mutation in *CASR*. The arrow indicates the mutation site (c.T1661A, *p.I554N*). **(B)** Schematic representation of human CASR structure and the corresponding site of *I554N* mutation (exon 6). **(C)** Conservation of the local amino acid sequence by multiple alignment of CASR from different species; *I554* is a highly conserved position. **(D)** *CASR I554N* mutation localized in the cysteine-rich region, which was critical for Ca^2+^ binding.

Direct sequence analysis of the relevant genes, including *CASR*, *GNA11*, and *AP2S1*, revealed a heterozygous missense T>A nucleotide substitution in exon 6 of the *CASR* gene at codon 1661. This substitution resulted in an amino acid change from isoleucine to asparagine (ATC to AAC, *I554N*) (**Figures 1A, B**). The missense *I554N* mutation was located in the cysteine-rich (CR) domain (residues 542–612) of the C-terminus of *CASR*. The mutated residue (*I554*) is highly conserved across different species (**Figure 1C**) and was not inherited from his parents. The *de novo I554N* mutation was not identified in 1517 healthy Taiwanese subjects according to the Taiwan Biobank database. Furthermore, it was predicted to be a pathogenic variant based on PROVEAN and Polyphen-2 scores.

### The effects of *CASR I554N* mutation on binding energy

**Figure 2:**
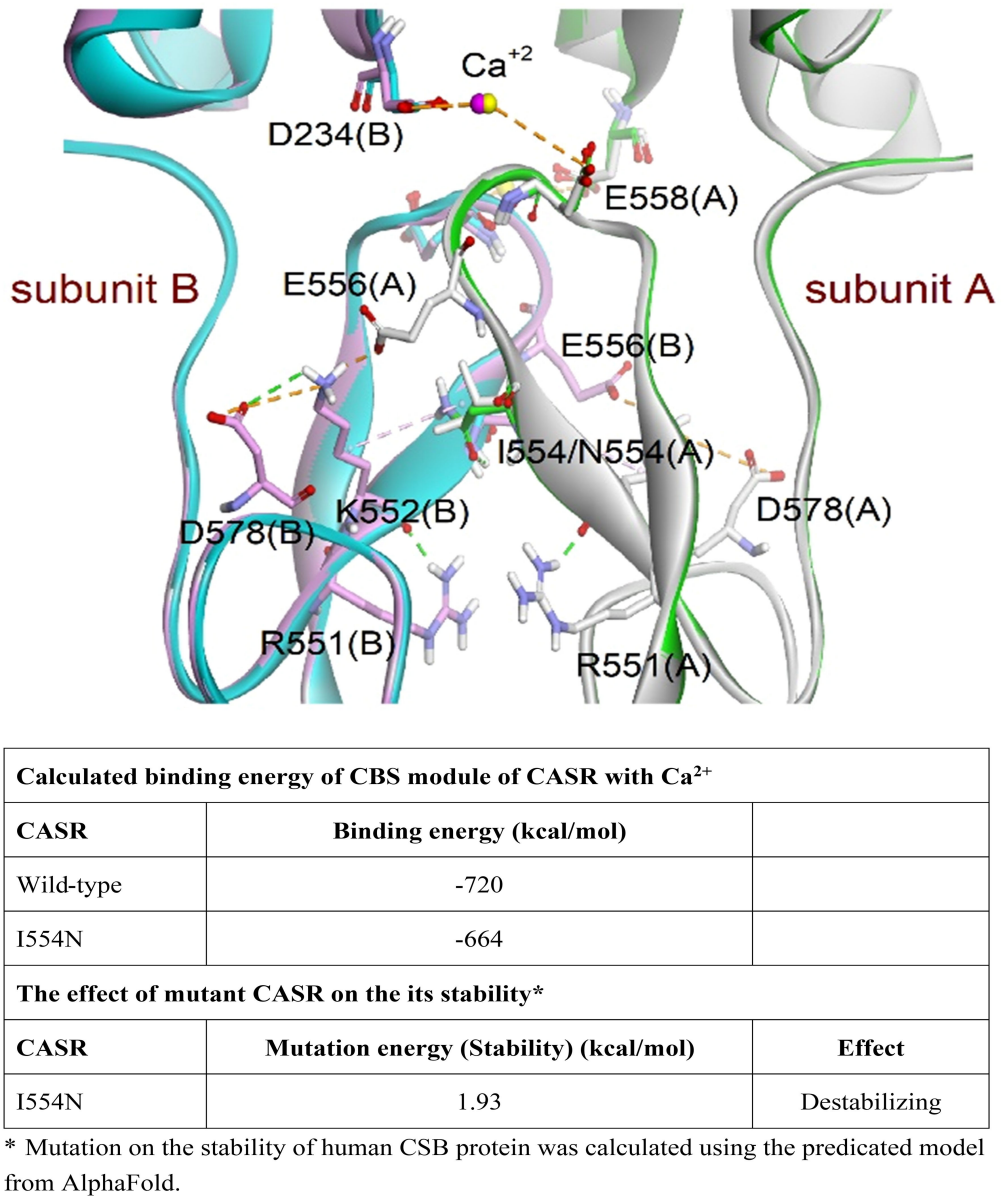
Simulation of the CASR models (PDB codes: 5K5S and 5K5T). Superimposition of wild-type (WT) and mutant CASR *I554N* models. The dimeric proteins were presented as a ribbon model and colored white and green for subunit A and pink and cyan for subunit B in WT and mutant CASR, respectively. The residues involved in interactions are shown as a stick model. The magenta and yellow spheres represent the calcium ions in WT CASR and mutant CASR, respectively. The hydrogen bond and electrostatic and hydrophobic interactions are shown as dashed green, orange, and pink lines, respectively. The *CASR I554N* mutation showed decreased binding ability to eCa^2+^ and its stability based on the CBS module and AlphaFold model.

According to The Human Gene Mutation Database in April 2023, 399 *CASR* mutations causing FHH1 have been reported, with the majority (88.1%) being missense/nonsense mutations. However, the pathogenic mechanism of the newly identified missense *CASR I554N* mutation has not been thoroughly investigated *in vitro*, and the therapeutic response to calcimimetic treatment has not been validated in affected patients. Fundamentally, *CASR I554* was localized in the CR region (**Figure 1D**; **Supplementary Figure 1**), which may impact CASR signal transduction (21). In line with this, the simulation model predicted that the *I554N* mutation affects the binding energy between CASR and Ca^2+^ (*WT*, -720 kcal/mol vs. *I554N*, -664 kcal/mol) (**Figure 2**). The impact of FHH1-associated mutations on the stability of our predicted CSB structures was calculated using BIOVIA Discovery Studio 2019 software. As for the calculation of the change in mutation, the corresponding mutation energy was normalized by CASR-WT protein. The result showed that *I554N* mutation exerted a destabilizing effect on Alphafold’s model (**Figure 2**).

### The instability of *CASR I554N* protein and decreased p-ERK1/2 levels

**Figure 3:**
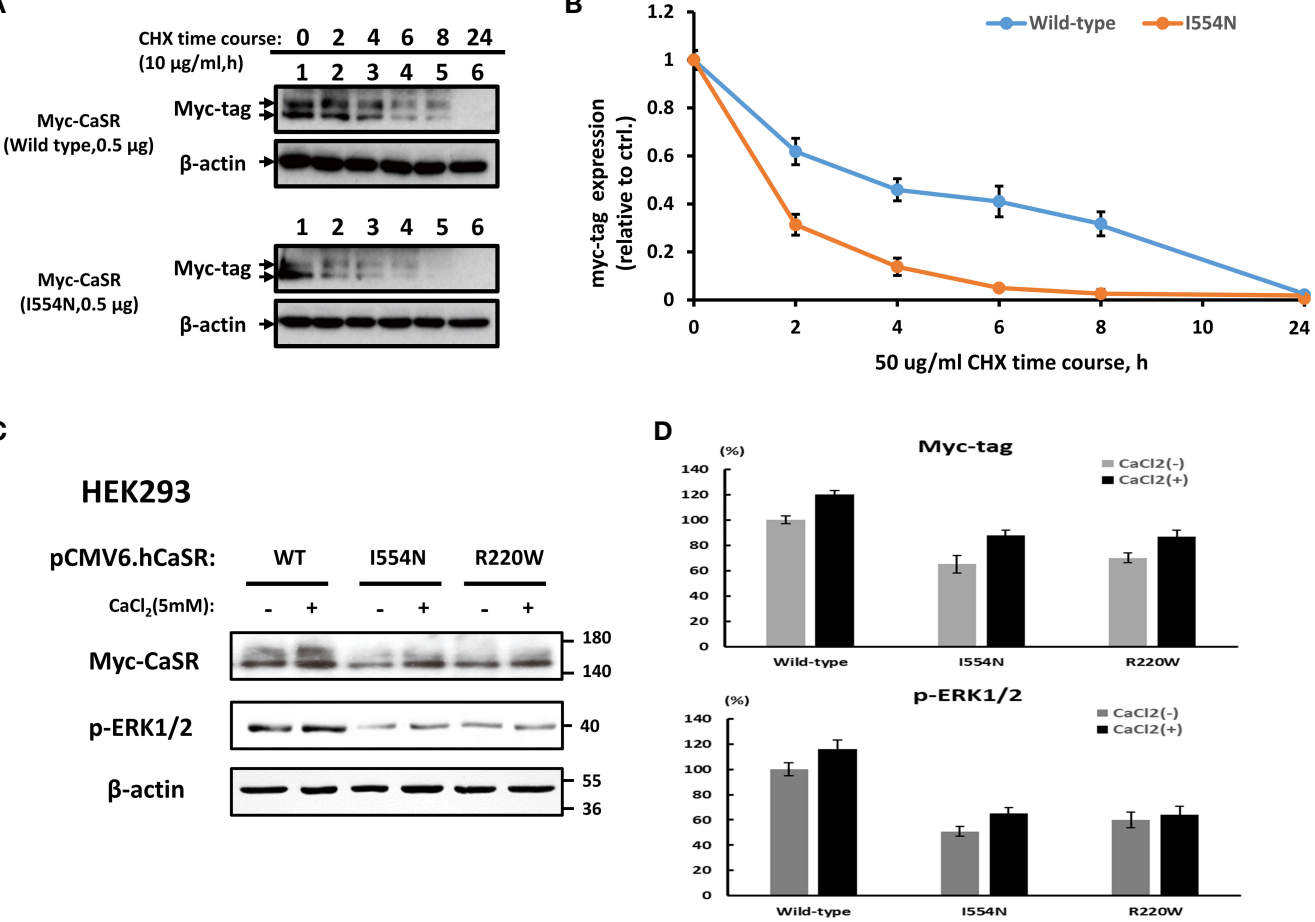
Analysis of the stability of CASR proteins and downstream p-ERK1/2 levels. A cycloheximide (CHX) chase study. **(A)** CASR protein expression was examined at different time points in HEK-293 cells transfected with CASR-**WT**-Myc and CASR-**I554N**-Myc vectors. **(B)** Representative quantified data expressed as the CASR/β-actin ratio. Results were normalized to β-actin control levels and expressed as the ratio change based on the level at time zero, which was set to 1.0. **(C, D)** Representative immunoblots and densitometry plots. **(C)** Representative IB analyses of the levels of CASR and p-ERK1/2 proteins in the HEK-293 cells transfected with CASR-**WT**-Myc, CASR-**I554N**-Myc, and CASR-**R220W**-Myc (inactivating CASR mutant: positive control) vectors. Compared with CASR-WT, the *CASR I554N* and *CASR R220W* mutations exhibited reduced CASR and p-ERK1/2 protein levels under basal and Ca^2+^-stimulated conditions. **(D)** The densitometry plots reflect the results of semi-quantification by densitometry (expressed as percentages and means ± SDs). Mouse Anti-Myc monoclonal antibody (TA150121-1; OriGene) was used to detect CASR expression in **(A, C)**.

To substantiate the above prediction, we generated mutant *CASR* constructs of interest for subsequent *in vitro* functional studies, and they expressed well in HEK-293 cells (**Supplementary Figure 2**). The time-course of cycloheximide (CHX) chase analysis showed that CASR I554N protein was more unstable than CASR-WT (**Figures 3A, B**). In addition, we selected the previously reported *CASR R220W* mutation as a positive control. The R220 residue is positioned within the VFT domain of the CASR and is crucial for ligand binding and receptor activation (22). It has been demonstrated that the *CASR R220W* mutation hinders the normal conformational changes in the VFT domain upon Ca^2+^ binding, leading to decreased sensitivity to eCa^2+^ levels and impairing the transduction of Ca^2+^ signaling (22). In comparison with CASR-WT, the levels of CASR and p-ERK1/2 proteins, both basal and Ca^2+^-stimulated, were decreased in the *CASR I554N* and *CASR R220W* mutations, indicating the abrogation of the MAPK pathway (**Figures 3C, D**).

### Blunt iCa^2+^ response to eCa^2+^ in the *CASR I554N* mutation

**Figure 4:**
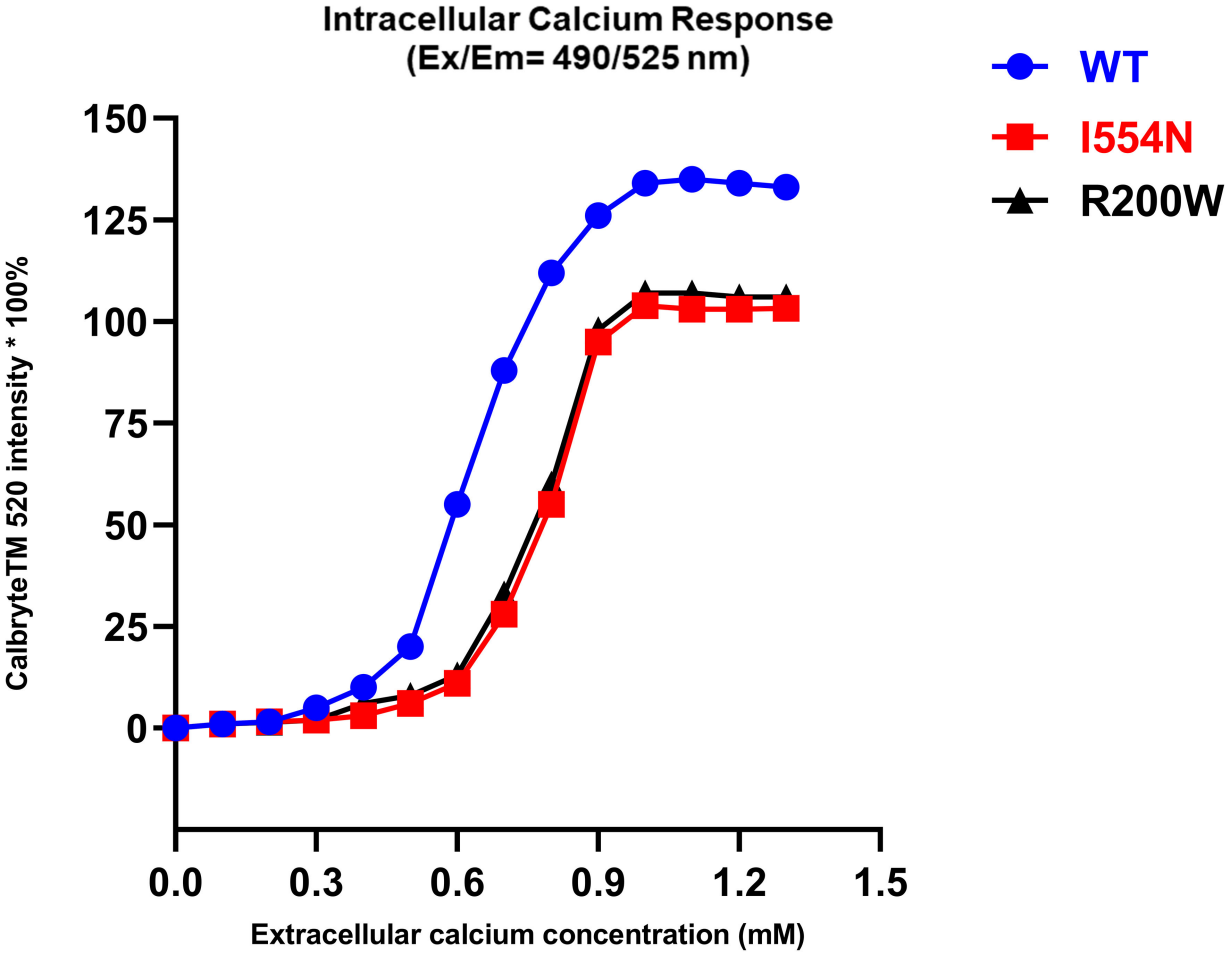
The intracellular calcium response in various CASR mutations. Calbryte™ 520 (Ex/Em=490/525 nm) is a new fluorescent and cell-permeable calcium indicator, which produces a bright fluorescence signal in the presence of iCa^2+^ at a high concentration. In contrast to CASR-WT, the CASR I554N mutation and the inactivating CASR R220W mutation exhibited a diminished response of iCa^2+^ to the increase in eCa^2+^ concentration.

CASR-WT showed a brisk intracellular Ca^2+^ (iCa^2+^) response after the eCa^2+^ concentration was slightly increased (0.2 mM), and a plateaued iCa^2+^ response was noted when eCa^2+^ reached 1.0 mM (**Figure 4**). In contrast, both the *CASR I554N* mutation and inactivating *CASR R220W* mutation exhibited a blunt response to the addition of eCa^2+^, indicating the inactivating function of the *CASR I554N* mutant.

### Calcimimetics rescue CASR function impaired by the *I554N* mutation

**Figure 5:**
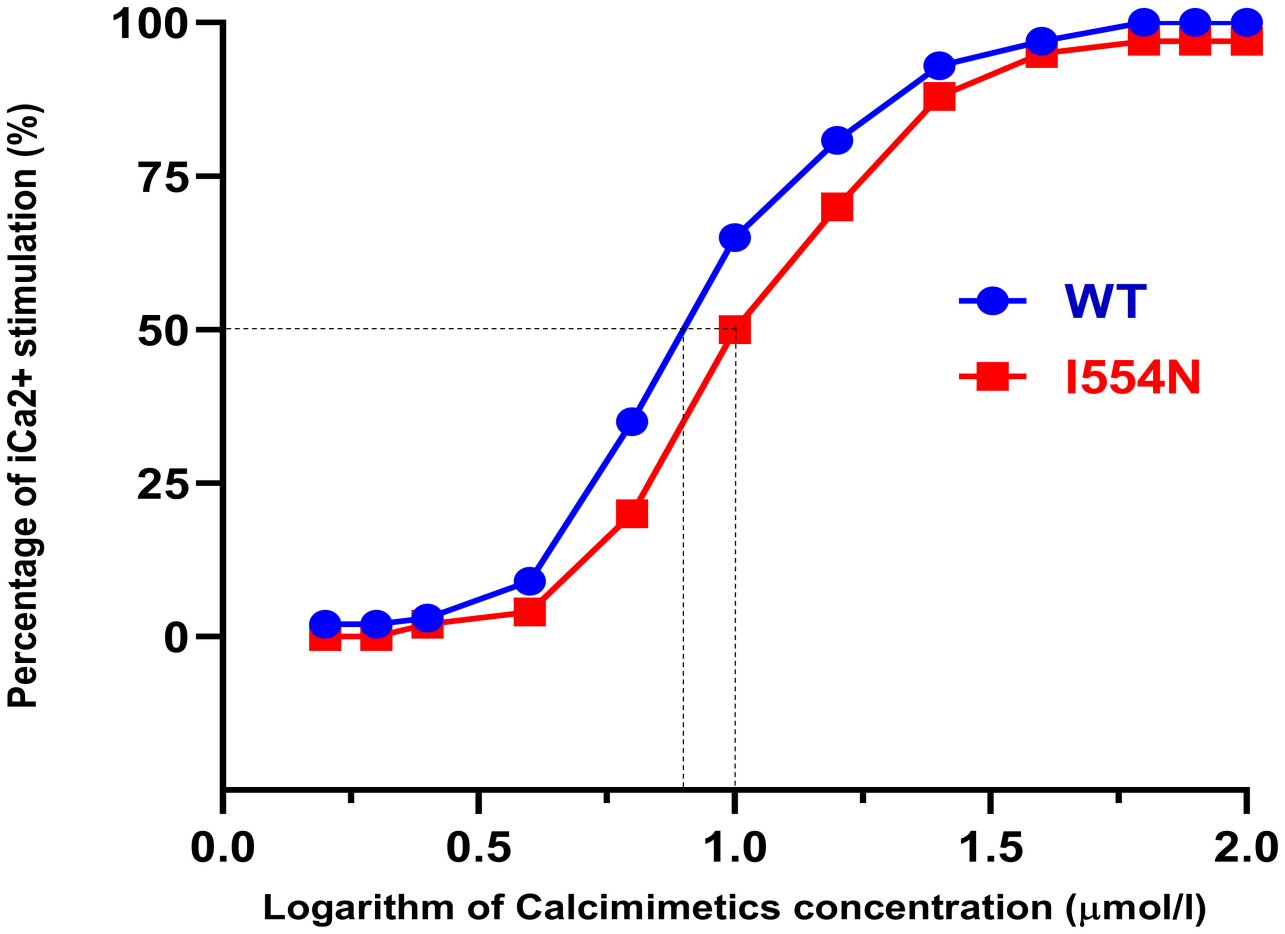
Response to calcimimetic NPS R-568 in CASR-WT and CASR-I554N. Calbryte™ 520 intensity [Percentage of iCa^2+^stimulation (%)] was examined at different calcimimetic NPS R-568 concentrations in HEK-293 cells transfected with CASR-**WT**-Myc and CASR-**I554N**-Myc vectors. HEK-WT and HEK-I554N cells were loaded with Calbryte™ 520 and stimulated by increasing NPS R-568 concentrations in the presence of 0.6  mmol/l CaCl_2_. The response was normalized and the percentage of iCa^2+^ stimulation was plotted against the logarithm of NPS R-568 concentrations. The dotted lines indicate the logarithmic values of NPS R-568 EC_50_.

As the simulation model showed that *I554* is localized in the CR region, the issue of whether calcimimetics (positive allosteric modulators) can rescue the dysfunction of *CASR I544N* should be clarified. Therefore, a fluorescence-based assay of Calbryte™ 520 was used to detect iCa^2+^. HEK-*CASR* WT and HEK-*CASR I554N* cells were stimulated with increasing concentrations of calcimimetic NPS R-568 (ranging from 1.0 to 100  μmol/l, **Figure 5**) in the presence of the same CaCl_2_ concentration (0.6  mmol/l). There was no significant difference in NPS R-568-EC_50_ between HEK-*CASR I554N* and HEK-CASR WT cells (NPS R-568-EC_50_: 10.02± 0.27 μmol/l vs. 7.10 ± 0.18 μmol/l, respectively), indicating calcimimetics can correct CASR function impaired by the *I554N* mutation. These findings also support the clinical presentation of our case, which showed an excellent therapeutic response to cinacalcet and the rapid correction of hypercalcemia within 6 months.

## Discussion

### The main findings regarding the *CASR I554N* mutation

**Figure 6:**
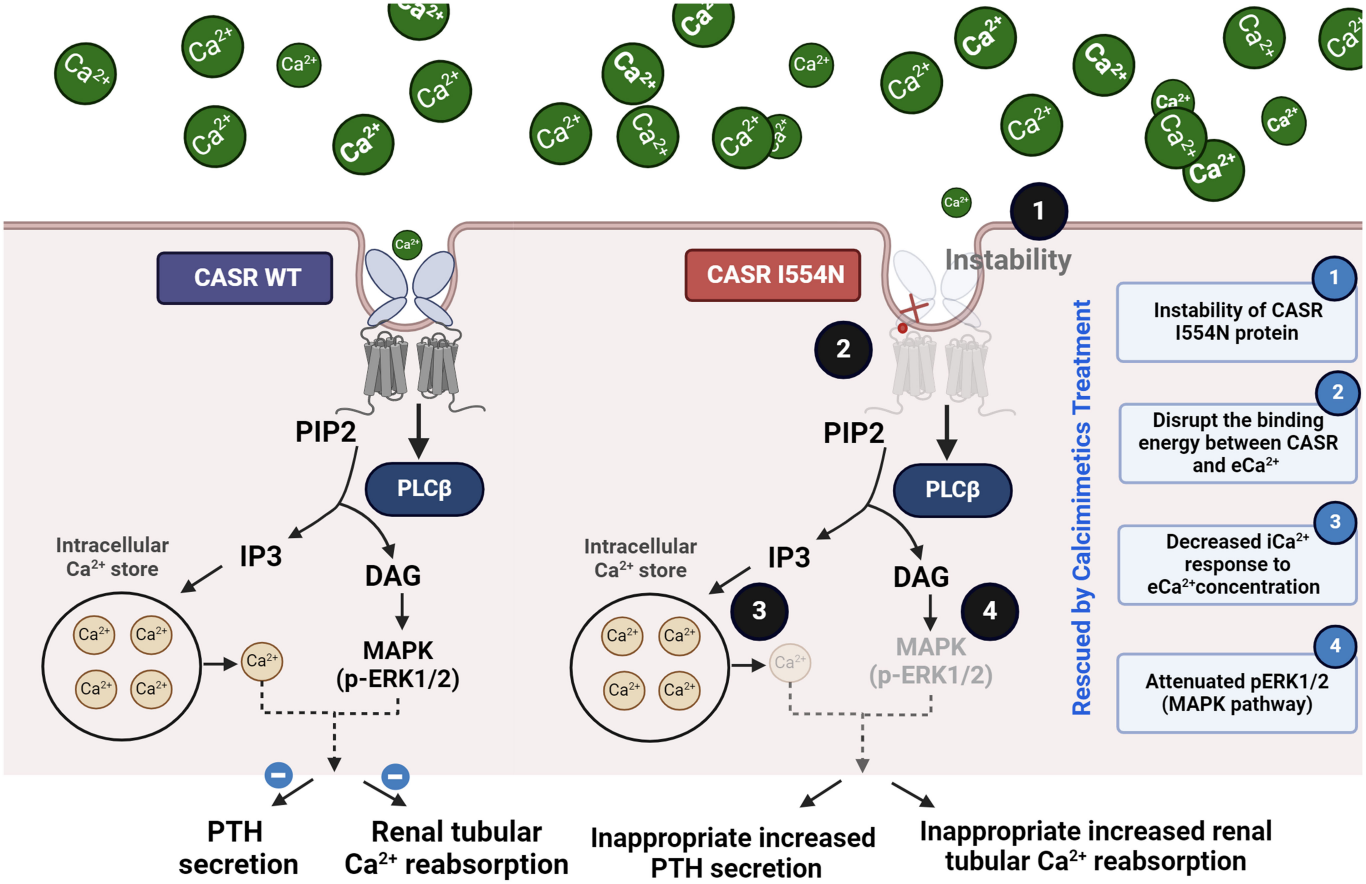
Summary of the mechanism of the pathogenic *CASR I554N* mutation.

A *de novo* heterozygous *CASR I554N* mutation was identified in our FHH1 patient. Simulation models showed decreased binding energy between the mutant *CASR I554N* and Ca^2+^, supporting pathogenic predictions based on PROVEAN and PolyPhen-2 scores. The *CASR I554N* mutation exhibited protein instability and reduced pERK1/2 expression, suggesting it could abrogate the MAPK pathway (**Figure 6**). The notion of defective CASR I554N protein was further supported by a decreased iCa^2+^ response to the gradient eCa^2+^ concentrations in a Calbryte 520 staining study. *In vitro* EC_50_ analysis revealed that the mutant *CASR I544N* responded well to the calcimimetic compound NPS R-568, restoring its function to a level comparable with *CASR WT*. These findings consistently demonstrated that our patient carrying the *CASR I554N* mutation presented with a dramatic response of cinacalcet to correct hypercalcemia in a short time.

### Decreased expression and stability of CASR and disruption of the downstream MAPK pathway

*In vitro* study showed that the *I554N* mutation contributed to the decreased CASR expression and the instability of CASR protein. It was reported that approximately 50% of the *CASR* mutations associated with FHH1 can reduce CASR expression due to defective trafficking to the plasma membrane (5, 15, 23). In short, mutant CASR is often retained intracellularly and is unable to exit the endoplasmic reticulum or Golgi apparatus (5, 15, 23). These previous findings align with our results, which demonstrate that the I554N mutation attenuates CASR protein expression and its downstream substrate phosphorylated ERK1/2, leading to a reduction in CASR function through the MAPK pathway (15). In addition to unstable expression, whether *CASR I554N* mutation can affect the binding affinity with eCa^2+^ should be investigated to gain new insight into the molecular mechanism of FHH1.

### The *I554N* mutation affected CASR dimerization and decreased the binding affinity to eCa^2+^

The human CASR is a dimeric cell-surface protein consisting of 1078 amino acids (5). It has a large ECD comprising 612 amino acids, which forms two globular lobes adopting a venus flytrap (VFT) conformation (5). A previous study examining FHH1-causing CASR mutations found that these mutations tend to cluster around the predicted calcium-binding sites, primarily located within the cleft region of the bilobed VFT domain (3). These mutated residues can directly disrupt the binding of eCa^2+^ or indirectly affect the conformational changes that occur upon eCa2+ binding, ultimately leading to the impairment of intracellular signaling cascades (3, 24–26). In our simulation model, the *CASR I554* mutation was observed to impact the dimerization structure of CASR. Additionally, the *CASR I554* mutation is situated in a cysteine-rich (CR) region, which potentially serves as an intramolecular switch regulating the entry and binding of eCa^2+^. Consistent with this notion, our *in vitro* study demonstrated that the *CASR I554N* variant exhibited a diminished response to increased eCa^2+^ concentrations, similar to the previously identified inactivating *CASR R220W* mutation (1).

### Limited calcimimetic functional study of missense CASR mutations

To date, FHH1 has been associated with 399 different mutations of *CASR*, with missense substitutions accounting for over 85% of cases, while nonsense, deletion, insertion, and splice-site mutations leading to truncated CASR proteins have been reported in less than 15% of cases (12, 27). Furthermore, *CASR* mutations have been found to cluster in three regions: the second peptide loop of the ECD, the VFT cleft region (eCa^2+^_o_-binding site), and the region encompassing transmembrane domains (TMD) 6 and 7 (5, 12). Intriguingly, FHH1 patients with *CASR* mutations in different domains exhibit distinct and variable phenotypic severities (1, 11). Although one *in vitro* study showed that calcimimetics can correct the expression in some *CASR* mutations (11), research on personalized treatment using calcimimetics for different CASR variants is limited (28–30). In addition, cinacalcet-unresponsive patients might harbor the missense mutations or in-frame deletions of *CASR* exon 5 encoding amino acids 460-536 in the extracellular domain (ECD) (31, 32), indicating that the different CASR mutations are likely responsible for the biodiversity in calcimimetics treatment. A better understanding of the effect of calcimimetics on heterogeneous FHH1 patients could greatly contribute to the development of novel therapeutics targeting the CASR-regulated MAPK pathway (33–36).

### A calcimimetic restored *CASR I554N* dysfunction in an EC_50_ study

Although FHH1 is considered the least severe form of FHH (3, 5–7), it still can result in unfavorable CKD or severe hypercalcemia associated with nephrolithiasis, for which surgical treatment is ineffective (9). To prevent these complications, calcimimetics may act as pharmacochaperones and provide a promising treatment option for FHH1 patients. They promote proper folding and/or increase the plasma membrane targeting of CASR mutants, as well as activate the CASR signaling pathway (MAPK pathway) (14, 23, 37–41). Of note, our *in vitro* study demonstrated that the EC_50_ value of NPS R-568 for the *CASR I554N* variant was similar to that of *CASR WT*, indicating that the calcimimetic corrected the dysfunction of the *CASR I554N* mutant by increasing iCa^2+^ mobilization (28). Although *CASR* mutations can exhibit different responses to calcimimetics due to the diverse types and locations of mutant variants in affected individuals, our study showed an excellent therapeutic response to the calcimimetic in our FHH1 patient. This suggests the calcimimetic could restore the function of the missense *CASR I554N* mutation localized in the CR region to a certain extent.

## Conclusion

Our study identified a *de novo* heterozygous pathogenic *CASR I554N* mutation that decreased CASR protein expression and stability, impaired binding to eCa^2+^, and attenuated pERK1/2 expression. The calcimimetics effectively corrected these dysfunctions *in vitro*. These findings have significant implications for FHH1 patients with CR region mutations, offering a practical approach to modulate CASR signal transduction.

## Data availability statement

The original contributions presented in the study are included in the article/**Supplementary Material**. Further inquiries can be directed to the corresponding author.

## Author contributions

C-ML: Conceptualization, Data curation, Formal analysis, Funding acquisition, Investigation, Methodology, Project administration, Writing – original draft. Y-XD: Data curation, Formal analysis, Investigation, Methodology, Writing – review & editing. S-MH: Supervision, Writing – review & editing. Y-CC: Supervision, Writing – review & editing. H-JL: Supervision, Writing – review & editing. C-CS: Writing – review & editing. S-HL: Supervision, Writing – review & editing.

## Supplementary material

Supplementary Figure 1 Summary of CASR pathogenic variants and corresponding locations among FHH1 patients. **(A)** The percentage of reported pathogenic variants in CASR, categorized by mutation types. **(B)** The percentage of missense/nonsense mutations in CASR, categorized by location. **(C)** The missense/nonsense mutations in CASR plotted according to their corresponding locations.

Supplementary Figure 2 Validation of mutant CASR construct transfection. Representative immunofluorescence micrographs of CASR proteins in the HEK-293 cells transfected with pCMV6-AN-mRFP-vector (negative control), pCMV6-CASR-**WT**-Myc (RC211229, OriGene), pCMV6-CASR-**I554N**-Myc, and pCMV6-CASR-**R220W**-Myc (inactivating CASR R220W: positive control). WT-CASR and mutant CASR constructs all expressed well in HEK-293 cells. Original magnification 400× **(A)** and 1000× (**B)**. Scale bars = 10 μm.
